# Early Video-assisted Thoracoscopic Surgery or Intrapleural Enzyme Therapy in Pleural Infection: A Feasibility Randomized Controlled Trial. The Third Multicenter Intrapleural Sepsis Trial—MIST-3

**DOI:** 10.1164/rccm.202305-0854OC

**Published:** 2023-10-11

**Authors:** Eihab O. Bedawi, Dionisios Stavroulias, Emma Hedley, Kevin G. Blyth, Alan Kirk, Duneesha De Fonseka, John G. Edwards, Eveline Internullo, John P. Corcoran, Adrian Marchbank, Rakesh Panchal, Edward Caruana, Owais Kadwani, Lawrence Okiror, Tarek Saba, Manoj Purohit, Rachel M. Mercer, Rhona Taberham, Nikolaos Kanellakis, Alison M. Condliffe, Leon G. Lewis, Dinesh N. Addala, Rachelle Asciak, Radhika Banka, Vineeth George, Maged Hassan, David McCracken, Anand Sundaralingam, John M. Wrightson, Melissa Dobson, Alex West, Graham Barnes, John Harvey, Mark Slade, Mae Chester-Jones, Susan Dutton, Robert F. Miller, Nick A. Maskell, Elizabeth Belcher, Najib M. Rahman

**Affiliations:** ^1^ Oxford Respiratory Trials Unit, Nuffield Department of Medicine,; ^2^ National Institute for Health and Care Research Oxford Biomedical Research Centre,; ^3^ Laboratory of Pleural and Lung Cancer Translational Research,; ^4^ Chinese Academy of Medical Sciences Oxford Institute, Nuffield Department of Medicine, and; ^5^ Oxford Centre for Statistics in Medicine, University of Oxford, Oxford, United Kingdom;; ^6^ Oxford Centre for Respiratory Medicine and; ^7^ Department of Cardiothoracic Surgery, John Radcliffe Hospital, Oxford University Hospitals National Health Service (NHS) Foundation Trust, Oxford, United Kingdom;; ^8^ Department of Infection, Immunity and Cardiovascular Disease, University of Sheffield, Sheffield, United Kingdom;; ^9^ Academic Directorate of Respiratory Medicine,; ^10^ Department of Thoracic Surgery, Northern General Hospital, Sheffield Teaching Hospitals NHS Foundation Trust, Sheffield, United Kingdom;; ^11^ School of Cancer Sciences, University of Glasgow, Glasgow, United Kingdom;; ^12^ Department of Respiratory Medicine, Queen Elizabeth University Hospital, Glasgow, United Kingdom;; ^13^ Department of Thoracic Surgery, Golden Jubilee National Hospital, Glasgow, United Kingdom;; ^14^ Department of Thoracic Surgery, Bristol Royal Infirmary, University Hospitals Bristol NHS Foundation Trust, Bristol, United Kingdom;; ^15^ Department of Respiratory Medicine and; ^16^ Department of Cardiothoracic Surgery, Derriford Hospital, University Hospitals Plymouth NHS Trust, Plymouth, United Kingdom;; ^17^ Department of Respiratory Medicine, Glenfield Hospital, University Hospitals of Leicester NHS Trust, Leicester, United Kingdom;; ^18^ Department of Thoracic Surgery, Glenfield Hospitals, University Hospitals of Leicester, Leicester, United Kingdom;; ^19^ Department of Respiratory Medicine and; ^20^ Department of Thoracic Surgery, Guy’s and St Thomas’ NHS Foundation Trust, London, United Kingdom;; ^21^ Department of Respiratory Medicine and; ^22^ Department of Cardiothoracic Surgery, Blackpool Teaching Hospitals NHS Foundation Trust, Blackpool, United Kingdom;; ^23^ Portsmouth Hospitals NHS Trust, Queen Alexandra Hospital, Portsmouth, United Kingdom;; ^24^ Department of Respiratory Medicine, PD Hinduja National Hospital, Mumbai, India;; ^25^ Department of Respiratory and Sleep Medicine, John Hunter Hospital, Newcastle, New South Wales, Australia;; ^26^ Hunter Medical Research Institute, Newcastle, New South Wales, Australia;; ^27^ Chest Diseases Department, Alexandria University, Alexandria, Egypt;; ^28^ Royal Victoria Hospital, Belfast Health and Social Care Trust, Belfast, Northern Ireland;; ^29^ Patient representative, Oxford, United Kingdom;; ^30^ Department of Respiratory Medicine, North Bristol NHS Trust, Bristol, United Kingdom;; ^31^ Academic Respiratory Unit, University of Bristol, Bristol, United Kingdom;; ^32^ Department of Respiratory Medicine, Gloucestershire Hospitals NHS Foundation Trust, Gloucester, United Kingdom; and; ^33^ Institute for Global Health, University College London, London, United Kingdom

**Keywords:** pleural empyema, video-assisted thoracic surgery, intrapleural, pleural effusion, randomized controlled trial

## Abstract

**Rationale:**

Assessing the early use of video-assisted thoracoscopic surgery (VATS) or intrapleural enzyme therapy (IET) in pleural infection requires a phase III randomized controlled trial (RCT).

**Objectives:**

To establish the feasibility of randomization in a surgery-versus-nonsurgery trial as well as the key outcome measures that are important to identify relevant patient-centered outcomes in a subsequent RCT.

**Methods:**

The MIST-3 (third Multicenter Intrapleural Sepsis Trial) was a prospective multicenter RCT involving eight U.K. centers combining on-site and off-site surgical services. The study enrolled all patients with a confirmed diagnosis of pleural infection and randomized those with ongoing pleural sepsis after an initial period (as long as 24 h) of standard care to one of three treatment arms: continued standard care, early IET, or a surgical opinion with regard to early VATS. The primary outcome was feasibility based on >50% of eligible patients being successfully randomized, >95% of randomized participants retained to discharge, and >80% of randomized participants retained to 2 weeks of follow-up. The analysis was performed per intention to treat.

**Measurements and Main Results:**

Of 97 eligible patients, 60 (62%) were randomized, with 100% retained to discharge and 84% retained to 2 weeks. Baseline demographic, clinical, and microbiological characteristics of the patients were similar across groups. Median times to intervention were 1.0 and 3.5 days in the IET and surgery groups, respectively (*P* = 0.02). Despite the difference in time to intervention, length of stay (from randomization to discharge) was similar in both intervention arms (7 d) compared with standard care (10 d) (*P* = 0.70). There were no significant intergroup differences in 2-month readmission and further intervention, although the study was not adequately powered for this outcome. Compared with VATS, IET demonstrated a larger improvement in mean EuroQol five-dimension health utility index (five-level edition) from baseline (0.35) to 2 months (0.83) (*P* = 0.023). One serious adverse event was reported in the VATS arm.

**Conclusions:**

This is the first multicenter RCT of early IET versus early surgery in pleural infection. Despite the logistical challenges posed by the coronavirus disease (COVID-19) pandemic, the study met its predefined feasibility criteria, demonstrated potential shortening of length of stay with early surgery, and signals toward earlier resolution of pain and a shortened recovery with IET. The study findings suggest that a definitive phase III study is feasible but highlights important considerations and significant modifications to the design that would be required to adequately assess optimal initial management in pleural infection.The trial was registered on ISRCTN (number 18,192,121).

**At a Glance Commentary**

**Current Scientific Knowledge on the Subject**

Combination intrapleural enzyme therapy with tissue plasminogen activator and DNase and surgical drainage using video-assisted thoracoscopic surgery are well-established techniques to manage nonresolving pleural infection in adults. These have never been prospectively and definitively compared head to head. There is also a growing body of evidence pointing toward delays in treatment contributing to ongoing poor outcomes.

**What This Study Adds to the Field**

This is the first multicenter randomized controlled trial of early intrapleural enzyme therapy versus early surgery in pleural infection. It demonstrates that a definitive trial is feasible, identifies interesting signals of potential advantages of early treatment using each modality, and provides valuable insight into the complexities of trial design in this area for future studies.

Pleural infection affects an estimated 80,000 patients annually in the United States and United Kingdom combined (1). The incidence has steadily increased (2–4), and clinical outcomes remain poor, with 30-day and 1-year mortality rates of 10% and 20%, respectively (5, 6).

The largest international multicenter prospective observational study to date Pleural Infection Longitudinal OuTcome (PILOT [6]) demonstrated that standard medical therapy with a chest tube and antibiotics fails in 33.5% of cases (6). Such patients are treated with one or both of two established treatment modalities: surgical intervention or combination intrapleural enzyme therapy (IET) with tissue plasminogen activator (tPA) and DNase.

Minimally invasive surgical techniques using video-assisted thoracoscopic surgery (VATS) have potentially widened the population suitable for surgical intervention. However, large case series (7, 8) demonstrate that patients undergoing surgery are consistently younger and have fewer comorbidities than unselected populations (9, 10). There are potentially significant numbers of patients in whom the potential of mortality from uncontrolled pleural sepsis may outweigh the risks of surgery and/or general anesthesia. Delays in surgical intervention are a predictor of conversion from thoracoscopic to open surgery (11, 12); it is therefore plausible that earlier surgical intervention may be beneficial. However, there remain no strong data to support the use of early surgery to improve key clinical outcomes. Two small randomized controlled trials (RCTs) of chest tube drainage versus surgery have demonstrated reduced length of hospital stay with initial surgical treatment (13, 14). However, these studies contained methodological issues, including an absence of standardized decision-making criteria, and have not altered practice.

Since the publication of the MIST-2 (second Multicenter Intrapleural Sepsis Trial) study, the use of IET has revolutionized medical management. Although MIST-2 was limited by a small number of patients in the IET arm (*n* = 52) and a primary outcome of radiographic clearance, multiple case series (15–19) comprising more than 600 patients have supported a reduced need for surgery and a shortened length of stay (LOS). A multicenter retrospective study of 1,850 patients treated with IET confirmed a low rate of bleeding complications (4.2%) and no major adverse events (20).

Thus, both surgery and IET appear to be effective interventions, and early introduction in treatment may improve outcomes. Direct comparison of early VATS and IET requires a phase III RCT; no such study has been conducted to date. The MIST-3 study was designed to assess the feasibility of early randomization to a surgical versus nonsurgical (i.e., IET) intervention and to specifically address the selection bias of previous studies (13, 14). The study aimed to randomize all participants enrolled, regardless of fitness for surgery, and sought to establish key outcome measures relevant to a subsequent definitive RCT. Information was collected on the feasibility of recruitment, participant acceptability, and the ability to collect outcome data.

## Methods

### Trial Design and Participants

MIST-3 was an open-label, multicenter, three-arm randomized controlled feasibility trial undertaken in eight centers in the United Kingdom. All eligible patients were included on screening logs, and the reasons for inclusion, exclusion, and/or randomization were recorded. The trial was registered on ISCRTN (number 18,192,121) and received ethical approval by the Cambridge East Research Ethics Committee (19/EE/0174).

Eligibility criteria were: *1*) clinical presentation compatible with pleural infection; *2*) pleural collection with a chest drain *in situ*; 3) pleural fluid on sampling that was macroscopically purulent, positive on Gram staining or culture for bacterial infection, or had a pH <7.2 (measured by blood gas analyzer) per previous studies (9, 10) and international guidelines (21, 22); *4*) evidence of residual collection and/or ongoing sepsis, including the presence of fever and increased serum levels of inflammatory markers such as C-reactive protein or an increased peripheral-blood white cell count, as assessed by the recruiting physician; and *5*) willingness to give written informed consent.

**Figure 1.**
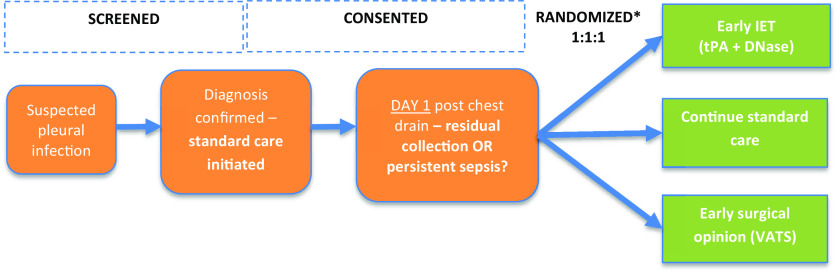
Trial design. IET = intrapleural enzyme therapy; RAPID = Renal (urea), Age, fluid Purulence, Infection source, Dietary (albumin); VATS = video-assisted thoracoscopic surgery. *Minimization for site and RAPID category

The exclusion criteria are outlined in the online supplement. Patients who met the eligibility criteria were screened and enrolled when the diagnosis had been confirmed. The date of chest tube insertion was considered to be trial Day 0. To exclude cases in which initial intervention resulted in complete pleural drainage, a run-in period of standard care (antibiotics and chest tube drainage) as long as 24 hours occurred after drain insertion. If a significant residual collection remained, the patient was eligible for randomization (confirmed by the local principal investigator [PI]) based on one or more predefined criteria of medical treatment failure (*see* online supplement) before randomization (trial Day 1) (Figure 1).

### Randomization

Eligible participants were randomized via an online randomization system (Sortition) on Day 1 on a 1:1:1 basis to continue standard care, receive early IET intervention, or receive a referral for early VATS. Randomization was stratified by center and baseline Renal (urea), Age, fluid Purulence, Infection source, Dietary (albumin) score (RAPID) risk score (6, 23) (Figure 1).

### Interventions

Complete details of intervention and treatment are provided in the online supplement. In brief, patients randomized to receive standard care were managed per current British Thoracic Society treatment guidelines (22). Patients assigned to undergo IET underwent treatment with intrapleural tPA (10 mg twice daily) and DNase (5 mg twice daily) through the chest tube (maximum of six doses over 72 h) (9). Treatment was started as soon as possible after randomization. Sites were able to reduce dosages of tPA on an individual case basis at the discretion of the local PI (18, 24).

Patients assigned to undergo surgery underwent surgical assessment by a local thoracic surgeon, and, if suitable, underwent surgery in accordance with the trial surgical standard operating procedure (*see* online supplement). The decision to proceed to surgery was at the discretion of the local surgical team.

#### Compliance and cross-over

Noncompliance with protocol treatment in each of the three arms was defined as follows. For the standard care arm, participants who received IET or surgery during their hospital stay were considered noncompliant. For the IET arm, noncompliance was noted if treatment was abandoned for a reason other than the clinician deeming that the treatment had been completed successfully and no further doses were required. Finally, for the VATS arm, participants who did not receive surgery (with surgical evaluation intended <48 hr after randomization) were considered noncompliant.

For safety reasons, cross-over was permitted if a different intervention was deemed clinically necessary (at PI discretion) and the trial intervention could not be achieved within 48 hours of randomization.

### Outcomes

The primary outcome was assessment of the feasibility of randomizing participants to the three arms of the study using recruitment rate, retention rate, and the proportion of participants screened who consented to be randomized, according to predefined criteria (*see* online supplement).

Secondary outcomes included hospital LOS, frequency of readmission, requirement for repeat intervention, visual analog scale (VAS) scores of pain, and quality of life. Complete details are included in the online supplement.

### Data Collection

Baseline clinical data were collected at enrollment. Full details, including inpatient study interventions, are provided in the online supplement. LOS was calculated from the date of randomization to the date of the patient being medically fit for discharge. Deaths occurring before discharge were excluded from the analysis. Follow-up data were collected at 2 weeks and 2 months, with an optional 6-month follow-up to allow for monitoring of late effects of treatment in each arm.

Patient distress and anxiety was assessed using the Hospital Anxiety and Depression Scale (HADS). Health utility scores using the EuroQol five-dimension health utility index (five-level edition; EQ-5D-5 L) were assessed, and the International Physical Activity Questionnaire (IPAQ; scored in metabolic equivalent of task minutes) was checked from raw values at baseline and 2 weeks, 2 months, and 6 months of follow-up. Pain scores were measured using a 100-mm VAS (25). Further details on the tools used and data collection are provided in the online supplement.

### Statistical Analysis

Because this was a feasibility study, a formal sample size was not calculated. A total of 75 patients were planned to be randomized (25 in each arm) over a period of 18 months from six centers based on recruitment to an observational study in pleural infection (PILOT), which recruited 20 participants per month in 20 centers (6).

To assess feasibility, the proportion of eligible participants was compared with the total number of patients screened, and the proportion of participants who consented to randomization was compared with the total number eligible. The proportion of patients who became ineligible as a result of good initial response to standard care (therefore not meeting the criteria for medical treatment failure at 24 h after chest tube insertion) and the recruitment and retention rates to discharge and 2 weeks were measured.

All patient-reported and clinical outcomes were analyzed on an intention-to-treat basis. Treatment difference and 95% confidence intervals (CIs) are reported throughout. Hospital LOS was summarized using a Kaplan-Meier plot, with deaths censored. Hospital LOS was defined from the date of randomization to the date of discharge. A mixed effects model adjusting for treatment, RAPID category, size of chest tube inserted, and baseline measurements as fixed effects and recruiting center as a random effect was fitted for continuous outcomes available at multiple time points.

Mean HADS score, EQ-5D-5 L utility index, EQ-5D-5 L 100-mm VAS score, and pain score were compared between groups using one-way ANOVA, with *post hoc* comparisons using the Tukey honestly significant difference test performed for statistically significant differences (*P* < 0.05).

### Impact of Coronavirus Disease (COVID-19)

The coronavirus disease (COVID-19) pandemic presented significant challenges. The first wave in the United Kingdom began in March 2020, 4 months after trial recruitment began (November 2019). The main impact was to trial recruitment rates, which, having been ahead of target, decreased substantially (Figure E1 in the online supplement). Based on a separate analysis of screening data, pleural infection rates in the United Kingdom decreased by approximately one third during the pandemic (26), with hospital admissions and research efforts predominantly COVID-19–related. Performing timely surgery and intervention in the context of COVID-19 became challenging as infection and prevention control measures became more restrictive and theater capacity was reduced. Further details of the impact of COVID-19 and mitigation strategies are outlined in the online supplement.

## Results

### Recruitment and Feasibility

**Figure 2.**
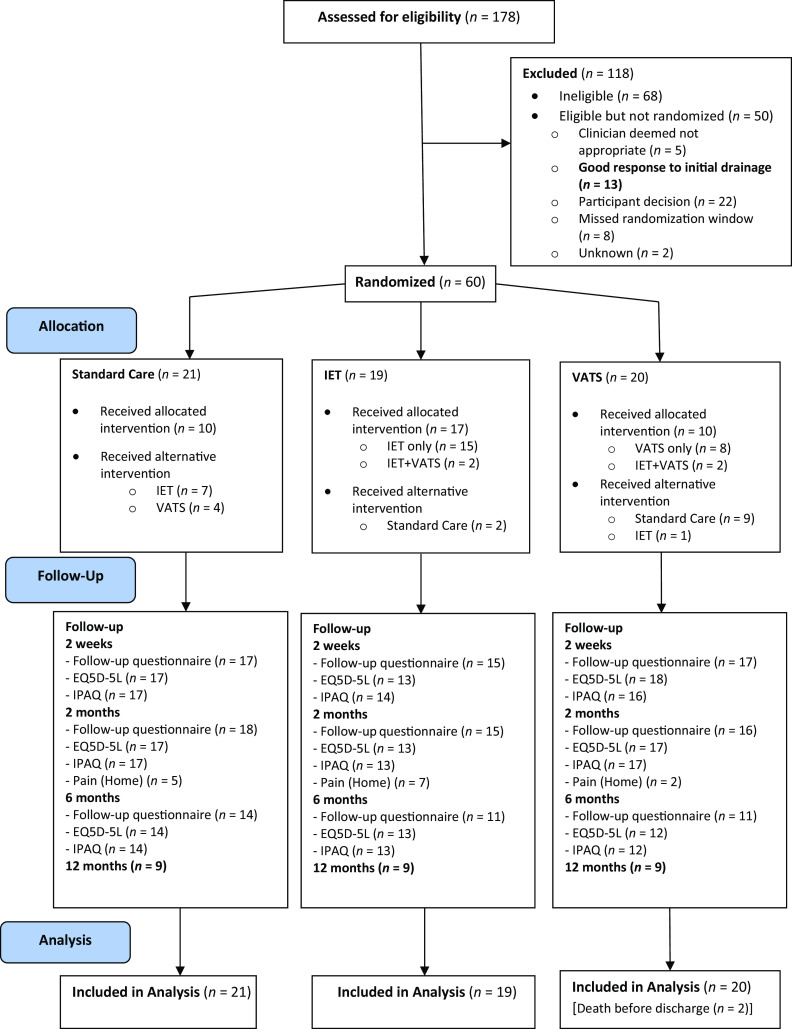
Consolidated Standards of Reporting Trials diagram from screening to analysis. EQ5D-5 L = EuroQol five-dimension health utility index, five-level edition; IET = intrapleural enzyme therapy; IPAQ = International Physical Activity Questionnaire; VATS = video-assisted thoracoscopic surgery.

Between November 1, 2019, and July 30, 2021, eight centers representing a geographical spread across the United Kingdom with a combination of on-site and off-site access to thoracic surgery services submitted screening logs for 178 patients. Of those screened, 110 patients met initial eligibility criteria; 13 of 110 patients (11.8%; 95% CI, 0.06–0.19) had a good response to initial treatment and were excluded from randomization. A total of 60 participants among the remaining 97 eligible participants were randomized (61.9%; 21 ongoing standard care, 19 early IET therapy, and 20 early surgical referral). All randomized participants were included in the analysis. The flow of participants through the study from screening to follow-up and availability of data are shown in Figure 2.

Baseline demographic, clinical, and microbiological characteristics were similar across all three groups (Table 1).

**Table 1.** Baseline Characteristics According to Treatment Group

Characteristics	Standard Care (*n* = 21)	IET (*n* = 19)	VATS (*n* = 20)
Age, yr	58 (51–72)	66 (56–71)	66 (59–74)
Male sex	13 (61.9%)	14 (73.7%)	11 (55.0%)
RAPID score			
0–2 (low)	9 (42.9%)	9 (47.4%)	9 (45.0%)
3–4 (moderate)	8 (38.1%)	7 (36.8%)	7 (35.0%)
5–7 (high)	4 (19.0%)	3 (15.8%)	4 (20.0%)
Comorbidities			
Respiratory disease	8 (38.1%)	4 (21.1%)	6 (30%)
Gastrointestinal	7 (33.3%)	7 (77.8%)	8 (40%)
Renal	2 (9.5%)	2 (10.5%)	2 (10%)
Cardiac	8 (38.1%)	10 (52.6%)	10 (50%)
Pleural fluid characteristics			
Purulence	10 (47.6%)	11 (57.9%)	7 (35%)
Culture positive	6 (28.6%)	4 (21.1%)	5 (25%)
Pleural fluid pH	6.98 (6.89–7.17)	6.90 (6.74–7.03)	7.03 (6.80–7.25)
Pleural fluid LDH, IU/L	1,160 (172–2,160)	1,650 (820–4,360)	1,660 (600–3,000)
Chest tube size			
12 F	17 (81.0%)	14 (73.7%)	18 (90.0%)
16 F	1 (4.8%)	0	0
18 F	3 (14.3%)	4 (21.1%)	2 (10.0%)
Other	0	1 (5.3%)	0

*Definition of abbreviations*: IET = intrapleural enzyme therapy; LDH = lactate dehydrogenase; RAPID = Renal (urea), Age, fluid Purulence, Infection source, Dietary (albumin); VATS = video-assisted thoracoscopic surgery.

Data presented as median (IQR) where applicable.

### Data Completion

Participant retention rate to hospital discharge was 100%. Two-week and 2-month follow-up completion rates were 84.5% and 87.5%, respectively (Table 2). Considering that the first wave of the COVID-19 pandemic occurred 4 months into trial recruitment, the 6-month follow-up was made optional and completion rates were removed from the analysis. There were two participant withdrawals, one from the standard care arm (patient choice) and one from the IET arm (lost to follow-up). Both withdrawals occurred after discharge.

**Table 2.** Feasibility Outcomes

Outcome	Value
Screened	178
Eligible	110
Not eligible as quick responder*	13
Quick responder rate	12% (6%–19%)
Actual eligible	97
Randomized	60
Randomization rate^†^	62% (54%–66%)
Survivors to discharge	58
Completed hospital discharge	58
Retention to discharge^‡^	100% (100%–100%)
Survivors to 2 wk	58
Completed Week 2 follow-up	49
Retention to 2 wk^§^	84% (73%–93%)

Data in parentheses are 95% confidence intervals.

* Responders are those screened but not eligible because they did not have a residual collection on Day 1 or C-reactive protein level decreased by more than half.

^†^ Randomized among actual number eligible.

^‡^ Number who completed hospital discharge/number of survivors.

^§^ Number who completed 2 weeks/number of survivors to 2 weeks after discharge.

The HADS was implemented during the inpatient phase 1–2 days after randomization, and the completion rate was 93% (20 of 21 [standard care], 18 of 19 [IET], and 18 of 20 [VATS]). At 2-week follow-up, completion rates of the IPAQ and EQ-5D-5 L questionnaire were 79% and 81%, respectively. Completion rates of both the IPAQ and EQ-5D-5 L questionnaire at 2 months and 6 months were 84% and 71%, respectively. Analysis was performed for the ITT population using available cases, with unacceptable values set to missing.

### Treatment Compliance and Cross-Over

Overall treatment compliance was 47.6% (10 of 21) in the standard care arm, 73.6% (14 of 19) for IET, and 50% (10 of 20) for VATS (Table 3).

**Table 3.** Compliance with Study Intervention

	Standard Care (*n* = 21)	IET (*n* = 19)	VATS (*n* = 20)
Received as randomized			
Yes	10 (47.6%)	12 (63.2%)	10 (50.0%)
No	11 (52.4%)	7 (36.8%)	10 (50.0%)
Treatment(s) received during hospital stay			
No VATS or IET	10 (47.6%)	2 (10.5%)	9 (45.0%)
IET	7 (33.3%)	15 (78.9%)	1 (5.0%)
VATS	4 (19.0%)	0	8 (40.0%)
IET + VATS	0	2 (10.5%)	2 (10.0%)

*Definition of abbreviations*: IET = intrapleural enzyme therapy; VATS = video-assisted thoracoscopic surgery.

All noncompliance in the standard care arm was due to clinician concern that the patient required further intervention. Seven patients received IET and four received VATS. Five cross-overs occurred in the standard care arm within 48 hours (four to IET and one to VATS) and were therefore classified as protocol deviations (Table 3).

Reasons for noncompliance in the IET arm included patient intolerance as a result of pain (*n* = 3), clinician-assessed bleeding risk (*n* = 1), and concern about subdiaphragmatic communication (*n* = 1) (Table 4). The mean number of IET doses received by participants randomized to the IET arm was 4.8 (standard deviation [SD], 1.4). Of the 59 doses of tPA administered in the study, 40 (68%) were 10 mg and 19 (32%) were 5 mg. Full details of IET dosing are presented in Table E2 in the online supplement.

**Table 4.** Reasons for Noncompliance with Intervention

Reason	Standard Care (*n* = 21)	IET (*n* = 19)	VATS (*n* = 20)
Patient intolerance	–	3 (15.8%)	0
Complications	–	0	0
Unavailability of staff to administer	–	0	0
Clinician choice	–	4 (21.1%)	0
Operator access	–	0	0
Theater access	–	0	0
Anesthetic risk deemed too high	–	0	2 (10.0%)
Clinician choice (improving/no longer required)	–	0	7 (35.0%)
Surgical capacity	–	0	1 (5.0%)
Not applicable	21 (100%)	12 (63.2%)	10 (50.0%)

*Definition of abbreviations*: IET = intrapleural enzyme therapy; VATS = video-assisted thoracoscopic surgery.

All patients in the VATS arm had a documented surgical evaluation within 48 hours of randomization. Analyzing reasons for noncompliance in the VATS arm (Table 4), 7 of 10 were deemed not to require surgery (i.e., clinical improvement) and did not receive further intervention before discharge. In 2 of 10 patients, the anesthesia risk was deemed too great, and these patients did not receive another intervention before discharge. One patient could not undergo VATS because of a lack of surgical capacity and crossed over to receive IET. Treatment compliance in the surgical arm was compared between recruitment sites with immediate access to surgeons on versus off site and was not shown to be different (Fisher exact test; 1 degree of freedom [df]; *P* = 0.53). No patients who received VATS required conversion to thoracotomy.

Two patients in the IET arm went on to receive VATS during hospital admission as a result of IET failure. Two patients in the VATS arm received IET while awaiting surgery. These patients were not classed as noncompliant.

### Time to Intervention

Median times to intervention were 1 day in the IET arm (IQR, 0–1) and 3.5 days for VATS (IQR, 1.2–4.0) (Mann Whitney *U* test, *P* = 0.02). In the IET arm, six patients commenced IET within 24 hours of randomization and only one patient took >48 hours to initiate therapy (*see* Table E2). In the VATS arm, 5 of 10 (50%) underwent an operation within 3 days of randomization, and 8 of 10 (80%) did so within 5 days of randomization (Table E3 in the online supplement).

### LOS

Overall median LOS across the study population was 9 days (IQR, 6–15). Median LOS according to RAPID category (low, moderate, high) were 6, 8.5, and 13 days, respectively (*P* = 0.032). Median LOS was 10 days (IQR, 7–13) in the standard care arm, 7 days (IQR, 5.5–10) in the IET arm, and 7 days (IQR, 5.5–10.5) in the VATS arm (*P* = 0.70). Further analysis showed no intergroup differences between individual arms (Table E4 in the online supplement).

### Further Intervention after Discharge

Further pleural infection–related admissions and interventions after discharge were analyzed. These were similar across the three treatment groups: 5 of 21 (23.8%) for standard care, 5 of 19 (26.3%) for IET, and 6 of 20 (30%) for VATS (χ^2^ = 0.20 [df = 2]; *P* = 0.90). Details are presented in Table 5.

**Table 5.** Further Admission and Surgery

	Standard Care (*n* = 21)	IET (*n* = 19)	VATS (*n* = 20)
Further hospital admission	5 (23.8%)	5 (26.3%)	6 (30.0%)
Further intervention			
Surgery	1 (4.8%)	0	1 (5.0%)
Chest drain	0	1 (5.3%)	1 (5.0%)
Other	1 (4.8%)	1 (5.3%)	0

*Definition of abbreviations*: IET = intrapleural enzyme therapy; VATS = video-assisted thoracoscopic surgery.

### Mortality

In the ITT analysis, the overall mortality rate at 12 months was 10% (6 of 60). Mortality showed a trend toward being higher in the VATS arm (4 of 20; 20%) compared with the standard care arm (1 of 21; 4.8%) and the IET arm (1 of 19; 5.3%) (χ^2^ = 3.33 [df = 2], *P* = 0.19). Two deaths occurred before discharge (2 of 60; 3.3%), both of which were in the VATS arm (Table 6). Deaths were analyzed per protocol because of the potential implications for the primary feasibility outcome. Patients in the IET and standard care arms who died had both received treatment as randomized. In the VATS arm, only one patient who died had received VATS (and died as a result of postoperative hemorrhage), with the remaining three not receiving any intervention beyond standard care, none of whom died directly as a result of untreated pleural sepsis (Table 7).

**Table 6.** All-Cause Mortality between Treatment Groups

Outcome	Standard Care (*n* = 21)	IET (*n* = 19)	VATS (*n* = 20)
Mortality	1 (4.8%)	1 (5.3%)	4 (20.0%)
Mortality before discharge	0	0	2 (10.0%)
Median (IQR) days on trial	59 (59–59)	174 (174–174)	58 (20–115)

*Definition of abbreviations*: IET = intrapleural enzyme therapy; VATS = video-assisted thoracoscopic surgery.

**Table 7.** Treatment Details for Participants who Died during the Trial

Pt. No.	Treatment Allocated	Treatment Received	Days on Trial	Follow-up at Time of Death	Final Cause of Death
1	VATS	No VATS or IET	23 d	Before discharge	Aspiration pneumonia
2	Standard care	No VATS or IET	59 d	2 mo	Septic shock secondary to community-acquired pneumonia on background of AML and CKD
3	IET	IET	174 d	6 mo	Natural death in nursing home
4	VATS	No VATS or IET	92 d	2 mo	Metastatic lung cancer
5	VATS	No VATS or IET	185 d	12 mo	Subdural hematoma
6	VATS	VATS	10 d	Before discharge	Large retroperitoneal hematoma secondary to hepatic artery rupture*

*Definition of abbreviations*: AML = acute myeloid leukemia; CKD = chronic kidney disease; IET = intrapleural enzyme therapy; VATS = video-assisted thoracoscopic surgery.

* Reported as a serious adverse event.

### Health Quality of Life and Physical Activity

Mean HADS score across the entire study at Day 1–2 after randomization was 16.7 (SD, 9.3; 95% CI, 14.2–19.2), with no differences between groups (Table E5 in the online supplement).

EQ-5D utility index scores range from 0 (equivalent to death) to 1 (representing perfect health). The IET arm showed the greatest improvement in mean EQ-5D utility index score from baseline to 2 months (from 0.35 to 0.83), compared with standard care (from 0.48 to 0.62) and VATS (from 0.38 to 0.59). Comparing the difference in the change in EQ-5D utility index scores between IET and VATS, this was statistically significant in favor of IET (*P* = 0.023) (Table 8).

**Table 8.** EQ-5D and Pain Scores

Score	Standard Care (*n* = 21)	IET (*n* = 19)	VATS (*n* = 20)
EQ-5D utility index*			
Baseline	0.485 ± 0.181	0.351 ± 0.157	0.382 ± 0.159
2 wk	0.629 ± 0.232	0.704 ± 0.223	0.591 ± 0.276
2 mo	0.616 ± 0.389	0.833 ± 0.126	0.587 ± 0.354
EQ-5D VAS score^†^			
Baseline	49.9 ± 20.7	45.1 ± 16.2	54.2 ± 23.8
2 wk	59.5 ± 29.1	67.7 ± 14.2	66.2 ± 17.0
2 mo	63.6 ± 25.8	79.5 ± 16.0	72.0 ± 19.2
Pain score			
After tube insertion	32.8 ± 16.8	36.4 ± 19.0	29.2 ± 14.5
In 2 mo after discharge	19.4 ± 8.2	4.9 ± 2.1	22.2 ± 9.5

Data presented as mean ± standard deviation.

*Definition of abbreviations*: EQ-5D = EuroQol five-dimension health utility index; IET = intrapleural enzyme therapy; VAS = visual analog scale; VATS = video-assisted thoracoscopic surgery.

* EQ-5D utility scores range from 0 to 1, with 1 representing perfect health and 0 equivalent to death. Participants who died before an EQ-5D measurement time point had their utility scores imputed as 0.

^†^ EQ-5D VAS reflects patient perception of overall health, and scores range from 0 (worst possible health) to 100 (best possible health).

The IET arm showed the greatest improvement in mean EQ-5D 100-mm VAS score for patient perception of overall health from baseline to 2 months (from 45.1 to 79.5), compared with standard care (from 49.9 to 63.6) and VATS (from 54.2 to 72.0). VATS did not show a benefit compared with standard care (*P* = 0.24), but there was a difference favoring IET compared with standard care (*P* = 0.027) (Table 8).

Inpatient mean pain scores were high across all interventions and were highest in the IET arm (mean, 36.4; SD, 19.0), followed by standard care (mean, 32.8; SD, 16.8) and VATS (mean, 29.2; SD, 14.5) (*P* = 0.89). At 2 months after discharge, mean pain scores were reduced in all groups; IET was associated with the lowest score (4.9; SD, 2.1), followed by standard care (19.4; SD, 8.2) and then VATS (22.2; SD, 9.5) (Table 8). The difference between groups did not meet statistical significance (*P* = 0.08).

### Per-Protocol Analysis

Because of variable compliance with the trial protocol, a per-protocol analysis of the main outcomes was performed (*n* = 35; standard care, *n* = 10; IET, *n* = 15; VATS, *n* = 10). The median LOS was 9.5 days (IQR, 4–16) in the standard care arm, 7 days (IQR, 5–12.5) in the IET arm, and 9.5 days (IQR, 7–17) in the VATS arm (ANOVA between groups, *P* = 0.47). Requirement for readmission and/or further intervention at 6-month follow-up was highest in the standard care arm at 5 of 10 patients (50%) and was similar in the IET arm (7 of 15; 47%), but was lowest among those who received upfront VATS at 2 of 10 patients (20%; χ^2^ test [2 df], *P* = 0.246) (Table E6 in the online supplement). EQ-5D utility index, EQ-5D 100-mm VAS score, and pain scores maintained similar intergroup differences between baseline and 2 months in favor of IET, but the differences were not statistically significant (Table E7 in the online supplement).

### Safety and Adverse Events

One serious adverse event occurred in a patient in the VATS arm who received surgery and died 10 days after randomization. The patient experienced acute kidney injury postoperatively with a decrease in hemoglobin level and was found to have a large retroperitoneal hematoma secondary to hepatic artery rupture. Most nonserious adverse events occurred in the IET arm, and the most common event was pain (Table 9).

**Table 9.** Adverse Events According to Treatment Received

	Standard Care (*n* = 21)	IET (*n* = 19)	VATS (*n* = 20)
Participants with probably or possibly related AEs*	1 (4.8%)	3 (15.8%)	0 (0%)
Pain	0	2	0
Acute kidney injury	1	0	0
Dizziness/presyncope	0	1	0
Other	0	1	0
Participants with unrelated AEs*	6 (28.6%)	7 (36.8%)	5 (25.0%)
Pain	1	4	1
GI upset (nausea/vomiting)	2	3	0
Transient delirium or confusion	1	0	1
Acute kidney injury	0	0	2
Swelling/erythema around drain site	0	0	1
Other	6	5	3

*Definition of abbreviations*: AE = adverse event; GI = gastrointestinal; IET = intrapleural enzyme therapy; VATS = video-assisted thoracoscopic surgery.

* Number of participants who reported at least one AE.

## Discussion

The treatment paradigm in pleural infection is based on expert consensus and has remained largely unchanged in the past two decades. The addition of combination intrapleural fibrinolytic and enzyme therapy has been a major advance. However, modern surgical techniques have meant that VATS has become a more accessible treatment for a significantly larger proportion of patients. Despite these developments, the two treatments have not been compared in a prospective multicenter study, resulting in variability in clinical practice and guideline recommendations (21, 22, 27). Outcomes in pleural infection remain unacceptably poor, and new treatment approaches are urgently needed.

In this study, the first head-to-head randomized study of IET versus surgery early in treatment, we demonstrated that patients presenting with pleural infection are amenable to early escalation to more “aggressive” therapies. After an initial period of standard care (chest tube drainage and antibiotics), using a protocolized definition of treatment failure, and despite a concurrent COVID-19 pandemic, 62% of eligible patients were successfully randomized to receive early IET or early surgical evaluation. Of the eligible patients who were not randomized, only 23% were not randomized as a result of direct participant refusal; hence, in general, there is equipoise and acceptance among clinicians and patients about participation in a surgery-versus-nonsurgery trial in pleural infection.

Although early cross-over (<48 h after randomization) was permissible if deemed clinically necessary, the high proportion of early cross-over in participants randomized to receive standard care likely represents a general trend toward early escalation among clinicians, suggesting that “standard care” has evolved ahead of guideline-driven practice. The degree of cross-over provides credence to the exclusion of a standard care arm in future trials in lieu of head-to-head randomization to IET and surgery alone. Furthermore, the observation that these patients were not heavily skewed toward one intervention (four received surgery, seven received IET) suggests reasonable equipoise among clinicians. The run-in period of initial treatment with antibiotics and a chest tube remains justified to exclude quick responders who may not require further intervention, who comprised approximately 13% of participants in the present study. This is an important finding and will inform future sample size calculations in a definitive trial.

In terms of the “active” interventions, treatment compliance was notably lower in the surgical arm (50%) compared with the IET arm (79%). The most common reason for patients not undergoing surgery was the risk/benefit balance of VATS no longer being in favor of proceeding with surgery by the time an operation was feasible. This should be considered when planning future phase III studies in which there is a large difference in delivering the trial intervention between two arms. Nonetheless, although most eligible patients agreed to be randomly allocated to surgery, and that anesthesia risk precluded only a minority of participants from this allocation, a minimum fitness criterion may potentially optimize compliance in a future phase III study while maintaining the strength of MIST-3 in avoiding the selection bias of previous surgical RCTs (13, 14), and the latter patients in their seventh and eighth decades of life were successfully treated surgically. With increasing experience, expertise, and safety demonstrated with VATS as a treatment modality, we strongly advocate that such patients are included in future trials given their increasing representation in pleural infection cohorts (28).

Adverse events related to treatment arm were minimal throughout the study (overall adverse events, 4 of 60; 6.7%). The most common AE was pain in the IET arm, which is well documented (20, 29, 30). It is noteworthy that, despite increased pain during administration, the reduction in mean pain score at 2 months in the IET arm was clinically significant (minimal clinically important difference of 16 mm) (31), which, when combined with the significantly favorable EQ-5D changes at 2 months compared with baseline, suggests that there are potentially important treatment effects in favor of IET that require further evaluation in a definitive phase III study.

A notable finding among the secondary outcomes concerned LOS. Despite median time to intervention varying significantly between IET and VATS (1 d vs. 3.5 d), it is of added value that these were similar in a recent U.S. pilot single-center RCT (32), reflecting generalizability across both healthcare systems. The median LOS was the same in both intervention arms at 7 days. Although MIST-3 was not powered to assess this outcome, this finding suggests that intervention at the earlier stages of the condition may be beneficial compared with the observed overall median LOS of 10–14 days in large studies (6, 9), and that an adequately powered RCT is needed to address this question.

Patient-reported outcome measures were a key focus in this study, as these have not been specifically evaluated in pleural infection. The HADS is a simple and effective measure of psychological and emotional distress (33). The study was not sufficiently powered to detect intergroup differences but brings to light the extent of the psychological impact of pleural infection.

The impact of the COVID-19 pandemic on the MIST-3 trial cannot be understated. The trial was recruiting well ahead of target until March 2020, when the first wave of the pandemic struck U.K. hospitals. Hospitalizations became predominantly COVID-19–related, and a substantial reduction in non–COVID-19–related admissions was observed across the Western world (34–36). Data from our own centers in a related study estimated that the incidence of pleural infection hospitalizations was reduced by approximately one third (26). The reasons for this are uncertain but likely attributed to the combined effect of shielding and isolation of vulnerable populations, reduced social mixing, widespread use of personal protective equipment, and liberal use of antibiotics to prevent secondary bacterial infections in patients with viral illness.

To our knowledge, MIST-3 is the first prospective multicenter study to successfully randomly assign patients with pleural infections to receive IET versus surgery. The results provide evidence for feasibility and acceptability, but, arguably, the study’s main success has been in identifying key aspects of study design and methodology that will inform future protocols for a trial of this kind. Strengths of the study include clear and standardized pleural infection diagnostic criteria and protocolized definition of medical treatment failure before randomization. Although the study is small and not powered to detect any treatment differences between groups, early intervention in general appeared to show a significant benefit in terms of LOS compared with standard care, and this finding adds credence to the need for a larger definitive study. Despite ITT analysis being the gold standard because it provides an unbiased estimate of treatment effect, its interpretation becomes difficult when a significant proportion of participants do not receive the intervention as randomized, as occurred in the surgical arm. We fully accept that a much larger study with longer follow-up would be needed to provide reliable evidence in regard to mortality, quality of life improvement, and long-term survival between IET and surgery. Based on the results of the MIST-3 study, a further definitive phase III study is being developed that does not include a standard care arm.

## Conclusions

This is the first multicenter RCT of early intrapleural enzyme therapy versus early surgery in pleural infection. It demonstrates the feasibility of recruitment and a potential shortening of LOS with VATS, but also signals toward earlier resolution of pain and return to usual function with IET. The study findings suggest that, with some modification to the trial design, a definitive phase III study is feasible and required to assess optimal initial management in pleural infection. Planning for this is under way.
